# Epithelial-mesenchymal transition gene signature is associated with prognosis and tumor microenvironment in head and neck squamous cell carcinoma

**DOI:** 10.1038/s41598-020-60707-x

**Published:** 2020-02-27

**Authors:** Ah Ra Jung, Chan-Hun Jung, Joo Kyung Noh, Young Chan Lee, Young-Gyu Eun

**Affiliations:** 1 Department of Otolaryngology - Head & Neck Surgery, School of Medicine, Kyung Hee University, Seoul, Korea; 2 Department of Biomedical Science and Technology, Graduate School, Kyung Hee University, Seoul, Korea

**Keywords:** Cancer genomics, Prognostic markers, Head and neck cancer

## Abstract

In this study we assessed the clinical significance of an epithelial-mesenchymal transition (EMT) gene signature and explored its association with the tumor microenvironment related to immunotherapy in patients with head and neck squamous cell carcinoma (HNSCC). Genes were selected when mRNA levels were positively or negatively correlated with at least one well-known EMT marker. We developed an EMT gene signature consisting of 82 genes. The patients were classified into epithelial or mesenchymal subgroups according to EMT signature. The clinical significance of the EMT signature was validated in three independent cohorts and its association with several immunotherapy-related signatures was investigated. The mesenchymal subgroup showed worse prognosis than the epithelial subgroup, and significantly elevated PD-1, PD-L1, and CTLA-4 levels, and increased interferon-gamma, cytolytic, T cell infiltration, overall immune infiltration, and immune signature scores. The relationship between PD-L1 expression and EMT status in HNSCC after treatment with TGF-β was validated *in vitro*. In conclusion, the EMT gene signature was associated with prognosis in HNSCC. Additionally, our results suggest that EMT is related to immune activity of the tumor microenvironment with elevated immune checkpoint molecules.

## Introduction

Epithelial-mesenchymal transition (EMT) refers to a process whereby the adhesive polarity of epithelial cancer cells dissipates and changes to mesenchymal cells. This occurs in conjunction with increased cell migration and invasiveness and is also known to play an important role in cytoskeletal remodeling and resistance to apoptosis^1^. Several studies have reported the association of EMT activation with cancer metastasis, resistance to anticancer drugs, and thus a poor prognosis^2–4^.

Head and neck squamous carcinoma (HNSCC) is the sixth most prevalent cancer worldwide, with mortality rates of 40–50% despite advances in radiation and surgical treatments^5^. Radiotherapy and cytotoxic chemotherapy for HNSCC are associated with substantial toxicity and morbidity. There is no biomarker that can predict response to treatments, such as radiotherapy, chemotherapy, and especially immunotherapy in patients with HNSCC. Immunotherapy has begun a new era in cancer treatment by using treatments such as checkpoint inhibitors that target the host immune system instead of tumors^6^. Immune checkpoint inhibitors have showed promising preliminary data and were approved for use by the FDA in patients with advanced HNSCC^7–9^. Few studies have reported the impact of EMT on the interactions between cancer and immune cells.

We sought to develop an EMT gene signature that can predict prognosis by systematically analyzing genomic data from several independent cohorts of patients with HNSCC. In addition, we analyzed the association between EMT gene signatures and several immunotherapy-related gene signatures with the aim of determining whether the activation status of EMT signatures corresponds to the tumor microenvironment related to immunotherapy.

## Results

### Discovery of EMT related gene signature in patients with HNSCC

We identified the genes where mRNA expression levels were significantly correlated with each EMT marker, namely E-cadherin (*CDH1*), vimentin (*VIM*), N-cadherin (*CDH2*), and fibronectin 1 (*FN1*). EMT related gene signature was developed and consisted of 82 genes correlated with the four EMT marker genes (Supplementary Table S1).

**Figure 1:**
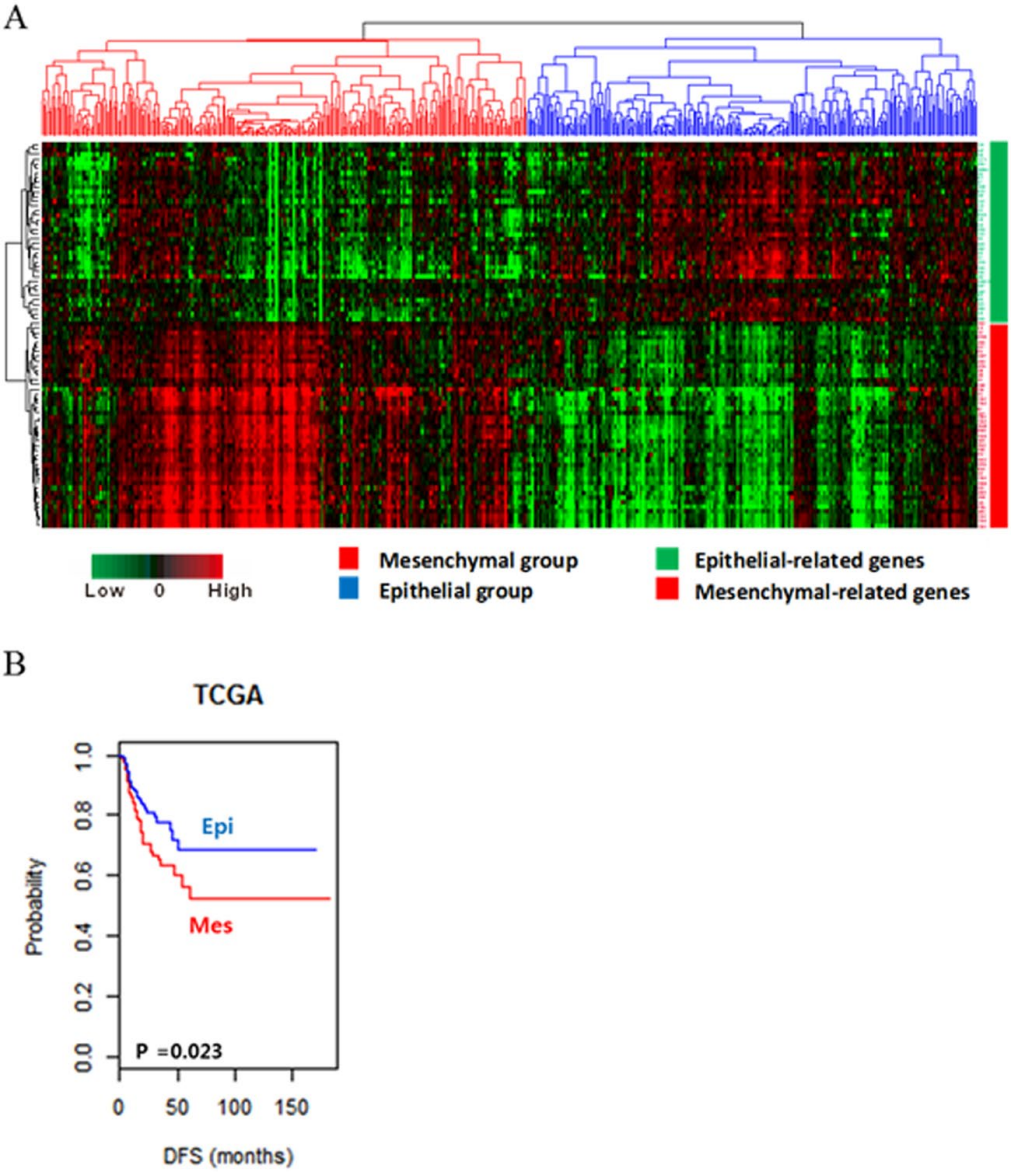
Stratification of patients with HNSCC in the TCGA cohort with EMT signature. (**A**) Patients in the TCGA cohort were clustered by two-way hierarchical clustering using the Pearson correlation distance between genes (rows), Euclidean distance between EMT signature genes (columns), and Ward’s linkage rule. The TCGA cohort was divided into two distinct subgroups of HNSCC: the Mes group (red) and Epi group (blue). The 82 EMT signature genes were separated into distinct epithelial-related genes (green bar) and mesenchymal-related genes (red bar). **(B**) Kaplan-Meier plots of DFS of patients with HNSCC in the TCGA cohort.

We identified two distinct subgroups of HNSCC - mesenchymal (Mes) and epithelial (Epi) subgroups – by hierarchical clustering of gene expression data of the training cohort (TCGA cohort). The genes of EMT signature were divided into distinct epithelial- and mesenchymal-related genes. The Mes subgroup had highly expressed mesenchymal-related genes, whilst the Epi subgroup had highly expressed epithelial-related genes (Fig. 1A). The Kaplan-Meier plots and the log-rank test showed that the disease-free survival (DFS) differed significantly between the two subgroups. The patients in the Mes subgroup showed worse DFS than those in the Epi subgroup (*p* = 0.02; Fig. 1B).

### EMT gene signature was associated with the prognosis of patients with HNSCC

**Figure 2:**
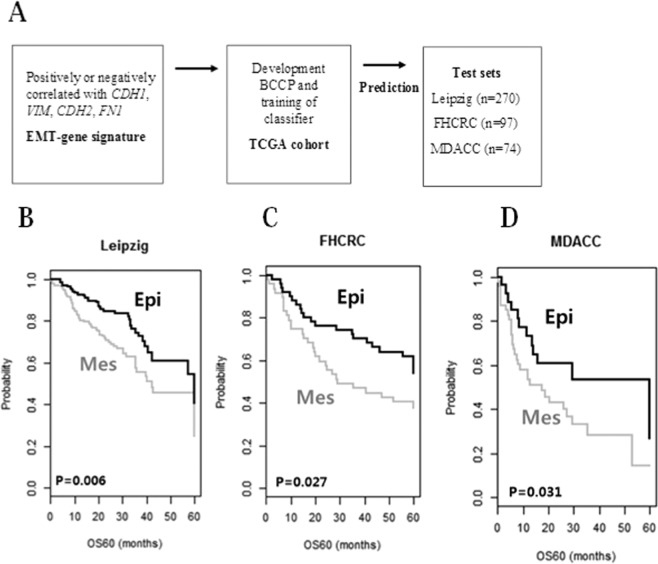
Construction of the prediction model and evaluation of the predicted outcome. (**A**) Schematic overview of the strategy used for constructing prediction models and evaluating the predicted outcomes based on gene expression signatures. (**B**) Kaplan-Meier plots of OS of the Leipzig cohort. (**C**) Kaplan-Meier plots of OS of the FHCRC cohort. (**D**) Kaplan-Meier plots of OS of the MDACC cohort. Patients were stratified by EMT signatures. The differences between groups were statistically significant, as indicated by the log-rank test.

Three independent cohorts (Leipzig, FHCRC, and MDACC cohorts) were used to validate the robustness of the EMT gene signature. Validation was performed as outlined in the flow chart in Fig. 2A.

Consistent with the results from the training cohort, patients were classified patients into Epi and Mes subgroups based on the EMT signature. When patients in the Leipzig, FHCRC, and MDACC cohorts were stratified according to EMT gene signatures, significant prognostic differences between subgroups were identified in all independent validation data sets. Five-year overall survival (OS) probabilities for subgroups were significantly different in all cohorts [Leipzig (*p* = 0.006; Fig. 2B), FHCRC (*p* = 0.027; Fig. 2C), and MDACC (*p* = 0.031; Fig. 2D) cohorts]. These results demonstrated the robustness of the prognostic value of EMT signature.

Univariate and multivariate analyses were performed on data from the TCGA, Leipzig, and MDACC cohorts (n = 825). These analyses were based on available clinical data to verify that the prognostic effect of EMT signatures acts independently of other clinical variables. The variables include patient age, gender, smoking status, alcohol consumption, anatomic site, HPV status, primary tumor stage, lymph node metastasis, and TNM stage. Gender, age, smoking status, alcohol consumption, anatomic site, HPV status, lymph node metastasis, and the EMT gene signature were statistically significant variables associated with OS in the univariate analysis. Alternatively, HPV status, lymph node metastasis, TNM stage, and EMT gene signature were statistically significant in the multivariate analysis. (Table 1).

**Table 1:** Univariate and multivariate Cox proportional hazard regression analysis of overall survival in the TCGA, Leipzig and MDACC cohorts (n = 825).

Variables	Univariate		Multivariate	
	HR (95% CI)	*p*-value	HR (95% CI)	*p*-value
EMT signature (Epithelial)	0.65 (0.52–0.83)	0.00046*	0.65 (0.50–0.84)	0.0013*
Gender (male)	0.76 (0.59–0.98)	0.037*	0.81 (0.60–1.11)	0.197
Age (>60 y)	1.33 (1.05–1.67)	0.015*	1.27 (0.98–1.64)	0.069
Smoking (No)	0.41 (0.19–0.90)	0.028*	0.53 (0.21–1.28)	0.16
Alcohol (No)	0.56 (0.38–0.82)	0.031*	0.45 (0.26–0.79)	0.056
Anatomic site (Oropharynx)	0.56 (0.32–0.95)	0.034*	0.74 (0.42–1.30)	0.301
HPV status (HPV-positive)	0.41 (0.26–0.63)	6.98e-05*	0.53 (0.31–0.90)	0.019*
Primary tumor (T3 & 4)	1.34 (0.81–2.20)	0.247	1.07 (0.51–2.24)	0.851
Regional lymph node (N+)	1.75 (1.34–2.28)	3.4e-05*	1.71 (1.26–2.33)	0.00054*
Stage (stage III & IV)	4.14 (0.58–29.57)	0.156	1.65 (1.00–2.71)	0.048*

### HPV-positive and HPV-negative patients show distinct EMT gene signatures

**Figure 3:**
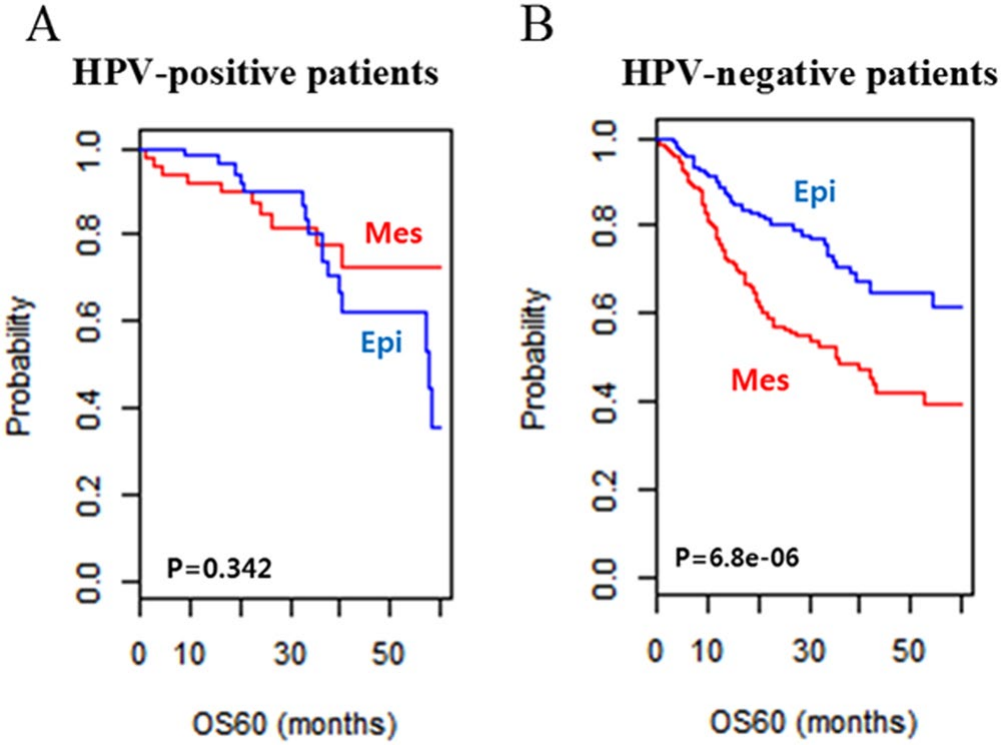
(**A**) Kaplan-Meier plots of OS of HPV-positive patients in the TCGA and Leipzig cohorts. (**B**) Kaplan-Meier plots of OS of HPV-negative patients in the TCGA and Leipzig cohorts. Patients were stratified based on EMT signatures. The differences between groups were statistically significant, as indicated by the log-rank test in HPV-negative patients.

The patients included in the TCGA and Leipzig cohorts were divided into HPV-positive and HPV-negative subtypes according to their HPV status. The survival analysis did not reveal statistically significant differences in 5-year OS between the Epi and Mes subgroups in HPV-positive patients (Log rank test *p* = 0.342; Fig. 3A). However, in HPV-negative patients, Mes subgroups had significantly worse 5-year OS than the Epi subgroup (*p* = 6.8e-06; Fig. 3B). These results suggest good predictability of the gene signature for HPV-negative patients.

### PD-1, PD-L1, and CTLA-4 levels were elevated in the Mes subgroup

**Figure 4:**
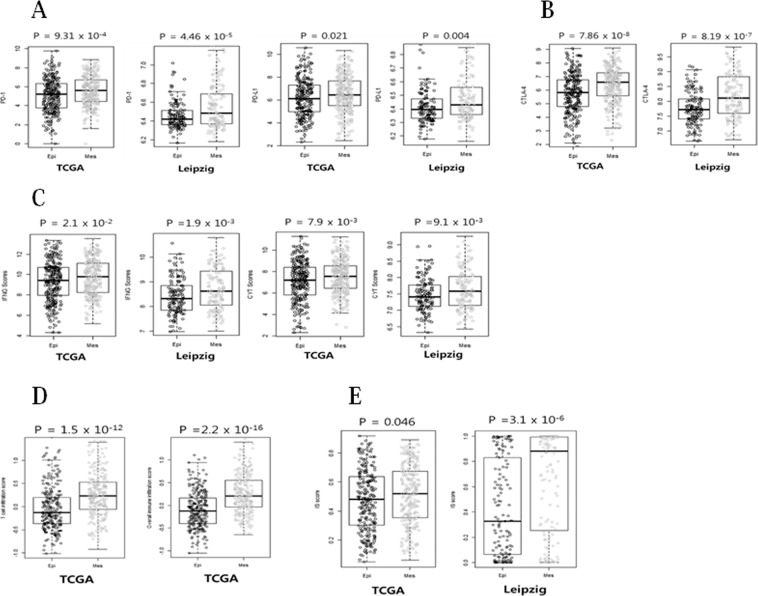
Evidence of immune checkpoint molecules and immune scores in Mes compared with Epi subgroups of HNSCC. (**A**) PD-1 and PD-L1 levels were significantly elevated in Mes subgroup as compared with Epi subgroup in the TCGA and Leipzig cohorts. (**B**) CTLA-4 level was significantly higher in the Mes subgroup than that in the Epi subgroup in the TCGA and Leipzig cohorts. (**C**) The INFG and CYT scores were analyzed in the Mes subgroup in comparison with the Epi subgroup of HNSCC patients for gene expression data from the TCGA and Leipzig cohorts. (**D**) TIS and IIS were analyzed in the Mes subgroup in comparison with the Epi subgroup of HNSCC patients for gene expression data from the TCGA cohort. (**E**) The IS score was significantly elevated in Mes subgroup as compared with Epi subgroup in the TCGA and Leipzig cohorts.

In many cancers, the PD-1 (PD-L1/PD-L2) pathway is known to be involved in tumor evasion from immune activity^10^. As shown in Fig. 4A, PD-1 (*PDCD1*) and PD-L1 (*CD274*) levels were significantly elevated in the Mes subgroup as compared with those in the Epi subgroup in the TCGA and Leipzig cohorts (*p* = 9.3e-4, *p* = 4.5e-5, *p* = 0.021, and *p* = 0.004, respectively).

Furthermore, CTLA-4 (*CTLA4*) expression was higher in the Mes subgroup than that in the Epi subgroup (Fig. 4B; *p* = 7.8e-8 and *p* = 8.2e-7, respectively). Notably, the level of immune checkpoint molecules, including CTLA-4 was significantly elevated in the TCGA and Leipzig cohorts.

### INFG, CYT, TIS, IIS, and IS scores were elevated in the Mes subgroup

To assess the association of EMT signature with immune activity in the tumor microenvironment we analyzed the gene expression of several immunotherapy-related scores. Interferon gamma (INFG) and cytolytic activity (CYT) scores were significantly elevated in the Mes subgroup in the TCGA and Leipzig cohorts (*p* = 0.021, *p* = 0.0019, *p* = 0.0079, and *p* = 0.0091, respectively, Fig. 4C). The T cell infiltration score (TIS) and overall immune infiltration score (IIS) were also significantly elevated in the Mes subgroup in the TCGA cohort (*p* = 1.57e-12 and *p* = 2.2e-16, respectively). Furthermore, the immune signature (IS) score was higher in the Mes subgroup in the both cohorts (*p* = 0.046 and *p* = 3.1e-6, respectively; Fig. 4E).

### TGF-β-induced EMT enhances PD-L1 expression in HNSCC cell lines

**Figure 5:**
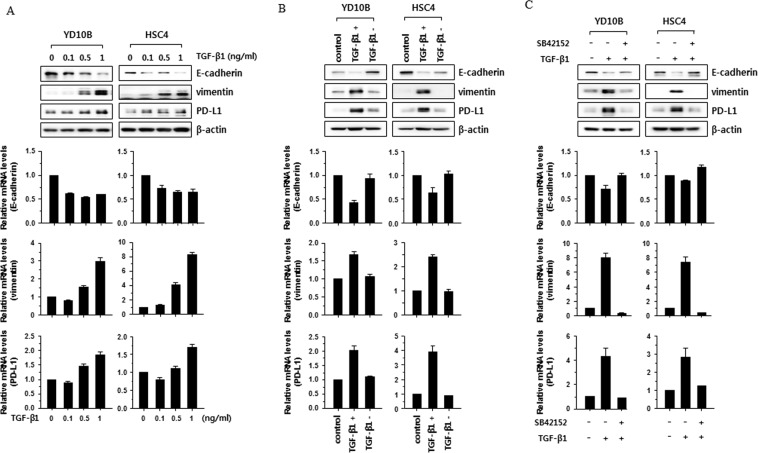
PD-L1 expression was enhanced by TGF-β-induced EMT. (**A**) YD-10B and HSC-4 cells were treated with the indicated concentrations of TGF-β1 (0.1, 0.5, and 1 ng/mL) for 2 days. (**B**) YD-10B and HSC-4 cells were incubated with medium containing 1 ng of TGF-β1 (TGF-β1 +) for 3 days, and then further incubated with medium without TGF-β1 (TFG-β1 –) for 5 days. (**C**) YD-10B and HSC-4 cells were treated with TGF-β1 (1 ng/mL) or TGF-β1 (1 ng/mL) plus SB 42152 (10 μM) for 2 days. (**A–C**) The levels of E-cadherin, vimentin, and PD-L1 were compared by western blotting (top) or real-time PCR (bottom). The real-time PCR data are presented as relative values normalized to those of the internal control (GAPDH). Full length uncropped western blotting images were shown in Supplementary Fig. S1.

The role of TGF-β as the primary inducer of EMT has been reported in several cancers^11–13^. Therefore, to investigate whether EMT status is associated with PD-L1 expression, we induced EMT status in YD-10B and HSC-4 HNSCC cells by treatment with TGF-β1 (0.1–1 ng/mL). To determine EMT status in YD-10B and HSC-4 HNSCC cells, expression levels of the epithelial cell marker (E-cadherin) and mesenchymal cell marker (vimentin) were analyzed using real-time PCR and western blotting. mRNA and protein levels of E-cadherin were decreased following treatment with TGF-β1 in a dose-dependent manner in YD-10B and HSC-4 cells, whereas mRNA and protein levels of vimentin were increased. Interesting, mRNA and protein levels of PD-L1 were increased (Fig. 5A). To determine whether PD-L1 expression was controlled by TGF-β-induced EMT, mRNA and protein levels of PD-L1 were analyzed in head and neck cancer cells under EMT and MET status using a reversion assay. mRNA and protein levels of PD-L1 were significantly altered under treatment with TGF-β1, as compared with control, and reverted to control levels after switching to culture medium without TGF-β1 (Fig. 5B). Furthermore, the TGF-β1-induced increase of PD-L1 expression was abolished by treatment with a TGF-β1 receptor kinase inhibitor (SB 431542) (Fig. 5C). These results suggest that PD-L1 expression is regulated by TGF-β-induced EMT status through the TGF-β signaling pathway.

### Association between EMT signature and clinicopathologic characteristics of HNSCC

We sought to identify the association between EMT signature and clinical or pathological characteristics of HNSCC, and hence we performed subset analyses in the TCGA and Leipzig cohorts (Supplementary Table S2). In particular, the association between EMT subgroups and tumor sites, T stage, regional lymph node metastasis, TNM stage, HPV status, smoking status, and alcohol consumption was evaluated. We did not find any significantly different clinical or pathological characteristics of HNSCC with which to identify the Mes and Epi subgroups.

### Relationship between EMT signature and somatic mutation

To identify somatic mutations that coincide with the activation of EMT signature in HNSCC, the somatic mutation data in the TCGA cohort (n = 493) were analyzed. Based on previous studies and results from the TCGA analysis, we selected six genes that are most likely to be associated with HNSCC^14–18^.

The somatic mutation frequencies of the six genes were compared between the Mes and Epi subgroups. The somatic mutation frequencies of *EGFR* and *TP53* were significantly different between the two groups (the Mes subgroup presented more frequent mutations) (Supplementary Table S3). There was no difference in mutation rates between the Mes and Epi subgroups for the other four genes.

### Biological process and pathway analysis

EMT signature gene analysis with DAVID bioinformatics resources 6.7 identified ten significant Kyoto Encyclopedia of Genes and Genomes (KEGG) pathways based on enrichment of mutations (Supplementary Table S4): ECM-receptor interaction (*p* = 4.3e-11), focal adhesion (*p* = 2.7e-10), protein digestion and absorption (*p* = 5.3e-8), amoebiasis (*p* = 4.0e-6), platelet activation (*p* = 1.3e-5), axon guidance (*p* = 0.016), and arrhythmogenic right ventricular cardiomyopathy (*p* = 0.035). Moreover, several pathways known to be important in cancer and HNSCC pathogenesis were identified, including the PI3K-AKT signaling pathway (*p* = 7.2e-7), pathway in cancer (*p* = 0.022), and microRNAs in cancer (*p* = 0.03).

## Discussion

In the present study, a robust EMT gene signature, clinically significant to patients with HNSCC, was developed and tested. The results revealed that Mes subgroup had poor OS in the training cohort. In the test cohorts, Mes subgroup showed worse OS and DFS. EMT signature showed independent prognostic effects in Cox proportional hazards analysis. Therefore, EMT gene signature may serve as a prognostic biomarker in HNSCC.

HPV is known to be involved in tumor formation through specific mechanisms, but it is unclear how this involvement affects EMT activation^19^. Jung *et al*. reported that HPV16 positivity correlates with the EMT transcription factor ZEB1 and that both E6 and E7 increase Slug, Twist, ZEB1, and ZEB2 expression^20^. Other studies have demonstrated that E6 or E7 inhibited E-cadherin at the cell surface^21^. In the present study, there was a significant difference in the OS of HPV-negative patients between the Mes and Epi subgroups, but not in the OS of HPV-positive patients. This suggests that EMT signature is another factor for predicting the prognosis of patients with HNSCC with respect to HPV status, which is line with a previous report showing that HPV positivity induces the expression of EMT-related genes^20,21^.

A number of authors reported that immune checkpoint inhibitors are effective in patients with elevated immune checkpoints, but biomarkers representing immune responses and immune checkpoint status are yet to be identified^22–24^. EMT is a biologic process related to decreased cell adhesion and increased invasiveness, and is known as a key program for metastasis and drug resistance in many cancers^2,3,25,26^. Studies have reported that EMT transcription factors (Snail or CCL2) can induce EMT process and are related to immunosuppressive cytokines activation and T-lymphocyte resistance in several cancers^27–30^. It has been reported that EMT can induce PD-L1 expression in non-small cell lung cancer^31^. However, the relationship between EMT and PD-L1 expression in HNSCC is not yet well known. We found that the levels of immune checkpoint genes, such as PD-1, PD-L1, and CTLA-4 were significantly elevated in HNSCC patients with “mesenchymal” phenotype. We also investigated whether EMT signature is closely associated with immune checkpoint genes in HNSCC cell lines. To study this, we induced EMT in HNSCC cell lines by treatment with TGF-β, and analyzed PD-L1 expression compared to cells under EMT and MET status. In this result, PD-L1 expression was enhanced by TGF-β-induced EMT, thereby indicating the relationship between EMT and immune checkpoint genes. Next, we sought to investigate whether EMT signature is associated with tumor microenvironment change using known immunotherapy-related gene signature. In the KEYNOTE-001 study, six gene signatures of INFG-related genes (IDO1, HLA-DRA, STAT1, CXCL9, IFNG and CXCL10) were previously developed in melanoma patients^32^. These six genes were significantly associated with the response to anti-PD-1 inhibitor in patients with melanoma. CYT was assessed by granzyme A (GZMA) and perforin (PRF1), and was dramatically associated with CD8 + T cell activation. Therefore, CYT could be used to predict clinical response to treatment with immune checkpoint inhibitors^33^. mRNA-based TIS was used for characterizing the computational immune cell decomposition for solid tumors. T cell infiltration in the tumor microenvironment is a key process in the balance of tumor and anti-tumor immune responses^33,34^. The IS score is known to be a transcriptional predictor of anti-CTLA-4 immune checkpoint inhibitor and was assessed in the genomic data from ~10,000 human tissues across 30 different cancer types to estimate the potential response to immunotherapy^35^. INFG, CYT, TIS, IIS, and IS scores were elevated in patients with “mesenchymal” phenotype as compared with those in patients with “epithelial” phenotype. Our findings demonstrated that EMT is associated with immune activation of the tumor microenvironment and elevation of multiple targetable immune checkpoint molecules, and may be used as a prognostic indicator in patients with HNSCC. EMT could induce cancer growth and metastasis by reprogramming immune activity in the tumor microenvironment. This suggests that patients in the Mes subgroup might have better response to immune checkpoint inhibitors, and targeting immune checkpoints may affect cancer metastasis and drug resistance associated with EMT. Therefore, our data suggest that EMT signature-based biomarkers may be valuable for identifying patients who can benefit from immune checkpoint blockade agents.

In conclusion, our results showed that the EMT signature was associated with the prognosis of patients with HNSCC, and in particular, patients in the Mes subgroup showed poor prognosis. In addition, there was an association between EMT and immune activity of the tumor microenvironment with elevated immune checkpoint molecules.

## Methods

### Patients and cohorts

For this study, clinical and gene expression data were collected from public databases representing four independent cohorts. Gene expression data of The Cancer Genome Atlas (TCGA cohort, n = 513) were downloaded from the UCSC Xena Browser (https://xena.ucsc.edu/)^18^. The data from the Institute for Medical Informatics, Statistics and Epidemiology (Leipzig cohort, GSE65858, n = 270)^36^, Fred Hutchinson Cancer Research Center (FHCRC cohort, GSE41613, n = 97)^37^, and MD Anderson Cancer Center (MDACC cohort, GSE42743, n = 74)^37^ were obtained from the National Center for Biotechnology Information Gene Expression Omnibus database (http://www.ncbi.nlm.nih.gov/geo) and used as the test sets. Gene expression data of TCGA and Leipzig were generated by Illumina HiSeq. 2000 and Illumina HumanHT-12 v4.0 Expression Beadchip, respectively. FHCRC and MDACC cohort data were generated by Affymetrix Human Genome U133 Plus 2.0 Array. Table 2 provides the details of the pathological and clinical characteristics of the patients in all cohorts.

**Table 2:** Patient demographics and clinical characteristics of all cohorts.

	TCGA cohort (N = 513)	Leipzig cohort (N = 270)	FHCRC cohort (N = 97)	MDACC cohort (N = 74)
Gender				
Male	370 (73.7%)	223 (82.6%)	66 (68.0%)	58 (78.3%)
Female	132 (26.3%)	47 (17.4%)	31 (32.0%)	16 (21.7%)
Age (mean ± SD)	60.9 ± 11.9	60.1 ± 10.0	NA	58.1 ± 13.4
Anatomic site				
Oral cavity	301 (60.0%)	83 (30.7%)	86 (88.7%)	71 (95.9%)
Oropharynx	79 (15.7%)	102 (37.8%)	11 (11.3%)	3 (4.1%)
Larynx	113 (22.5%)	48 (17.8%)	0	0
Hypopharynx	9 (1.8%)	33 (12.2%)	0	0
others	0	4 (1.5%)	0	0
Primary tumor				
T1	33 (6.8%)	35 (13.0%)	NA	3 (4.1%)
T2	147 (30.2%)	80 (29.6%)	NA	27 (36.4%)
T3	129 (26.5%)	58 (21.5%)	NA	28 (37.8%)
T4	178 (36.6%)	97 (35.9%)	NA	16 (21.7%)
Regional lymph node				
N0	238 (49.5%)	94 (34.8%)	NA	29 (39.1%)
N1	79 (16.4%)	32 (11.9%)	NA	13 (17.5%)
N2	155 (32.2%)	132 (48.9%)	NA	32 (43.4%)
N3	9 (1.9%)	12 (4.4%)	NA	0
Stage				
I	20 (4.1%)	18 (6.7%)	30 (30.9%)	3 (4.1%)
II	96 (19.6%)	37 (13.7%)	11 (11.3%)	16 (21.6%)
III	101 (20.7%)	37 (13.7%)	15 (15.5%)	15 (20.2%)
IV	272 (55.6%)	178 (65.9%)	41 (42.3%)	40 (54.1%)
HPV status				
Positive	68 (19.9%)	60 (23.4%)	0	NA
Negative	274 (80.1%)	196 (76.6%)	97 (100%)	NA
Tobacco use				
Never	114 (23.3%)	48 (17.8%)	NA	15 (20.3%)
Yes	376 (76.7%)	222 (82.2%)	NA	59 (79.7%)
Alcohol use				
Never	154 (42.1%)	31 (11.5%)	NA	NA
Yes EMT signature	212 (57.9%)	239 (88.5%)	NA	NA
Mesenchymal	266 (51.8%)	136 (50.3%)	47 (48.4%)	45 (60.8%)
Epithelial	247 (48.2%)	134 (49.7%)	50 (51.6%)	29 (39.2%)

Abbreviations: TCGA, The Cancer Genome Atlas; FHCRC, Fred Hutchinson Cancer Research Center; MDACC, MD Anderson Cancer Center; HPV, Human papilloma virus; EMT, Epithelial-mesenchymal transition; NA, not available.

### Development of EMT gene signature in HNSCC

The BRB-ArrayTools software program (http://brb.nci.nih.gov/BRB-ArrayTools/) was used for analysis of gene expression data^38^. The raw data were preprocessed using a robust multiarray averaging method for normalization^39^.

To find EMT-specific genes in HNSCC, gene expression data were analyzed from the TCGA cohort. Genes were selected when the mRNA expression levels were either positively or negatively correlated with at least one of the well-known EMT markers: E-cadherin (*CDH1*), vimentin (*VIM*), N-cadherin (*CDH2*), and/or fibronectin 1 (*FN1*). These markers were selected on the basis of their previously established roles as markers of EMT in lung cancer as well as other epithelial tumors^40^.

Using a gene feature and its correlated genes, hierarchical clustering analysis was performed with the centered correlation coefficient as the measure of similarity and a complete linkage clustering method. According to the patient clustering result, the patients were divided into two subgroups (mesenchymal and epithelial). Cluster analysis was performed with Cluster 3.0^41^.

### Construction of prediction models and validation in test cohorts

To test the ability of the gene expression signatures to predict the class of patients in an independent cohort, a previously developed model based on the Bayesian compound covariate predictor (BCCP) was adopted^42^. Gene expression data in the training set (TCGA cohort) were combined to form a series of classifiers according to the BCCP algorithm and the robustness of the classifier was estimated according to the misclassification rate determined during leave-one-out cross-validation of the training set. Validation was conducted in three independent patient groups (Leipzig, FHCRC, and MDACC cohorts).

### Immunotherapy-related gene score

To determine the association of EMT signature with immunotherapy, we used a previously described and validated immunotherapy-related gene signature. Interferon gamma (INFG) score was calculated as the average of the normalized values of the INFG-related six genes (*CXCL9*, *CXCL10*, *IDO1*, *IFNG*, *HLA-DRA*, and *STAT1)*^32^. The cytolytic activity (CYT) score was calculated as the average of the expression level of two key cytolytic effectors (*GZMA* and *PRF1*)^43^. The T cell infiltration score (TIS) was defined as the mean of the standardized values for all T cell subsets, except for T gamma delta and T follicular helper cells: CD8 T, T helper, T, T central and effector memory, Th1, Th2, Th17, and Treg cells^33^. The overall immune infiltration score (IIS) was defined as the mean of the standardized values for macrophages, dendritic cell subsets (total, plasmacytoid, immature, and activated), B cells, cytotoxic cells, eosinophils, mast cells, neutrophils, natural killer (NK) cell subsets [total, CD56(bright), and CD56(dim)], and all T cell subsets, excluding T gamma delta and T follicular helper cells^33^. The immune signature (IS) score was obtained using 105 immune signature genes^35^. The IS score of TCGA HNSCC cohort was obtained from the data of a previous study^35^.

### Pathway analysis

The list of EMT signature genes was submitted to the Database for Annotation, Visualization, and Integrated Discovery (DAVID) bioinformatics resources 6.7 to discover the gene ontology categories with significantly enriched gene numbers^44^. The default setting from the software was used to map the EMT signature genes to the reference set of direct and indirect relationships. Next, relevant inputs to the gene list, such as the molecular networks and biological functions were generated by the software’s algorithm. The significance of the gene annotation with a *p*-value less than 0.05 was determined with two-tailed Fisher’s exact test.

### Antibodies, recombinant protein, and inhibitor

The following antibodies were used in this study: anti-E-cadherin, anti-N-cadherin, anti-vimentin, and anti-PD-L1 from Cell Signaling Technology (Danvers, MA, USA); and anti-β-actin from Santa Cruz Biotechnology (Santa Cruz, CA, USA). Recombinant human transforming growth factor (TGF)-β1 was purchased from PeproTech (Rocky Hill, NJ, USA). SB 431542, a TGF-β inhibitor, was purchased from Tocris Bioscience (St. Louis, MO, USA).

### Cell culture and treatment

Human HNSCC, YD-10B, and HSC-4 cells were used in this study. YD-10B was purchased from Korean cell line bank (Seoul, Korea). HSC-4 was purchased from the Japanese Collection of Research Bioresources Cell Bank (Osaka, Japan). The cells were cultured in RPMI-1640 medium supplemented with 10% heat-inactivated fetal bovine serum. To induce EMT status in HNSCC, cells were incubated in the presence or absence of TGF-β1 (0.1–1 ng/mL) for 48 h. For reversion assay, cells were treated 1 ng/ml of TGF-β1 for 3 days, and then cultured in medium without TGF-β1 for 5 days.

### Western blotting

Cells were lysed on ice for 30 min in a buffer containing 20 mM Tris-HCl (pH 7.4), 100 mM NaCl, 0.5% NP-40, 0.1 mM Na_3_VO_4_, 50 mM NaF, 30 mM Na_4_O_7_P_2_ · 10 H_2_O, and a protease inhibitor cocktail (GenDepot, Barker, TX, USA). Equal amounts of protein in the lysates were separated by sodium dodecyl sulfate-polyacrylamide gel electrophoresis, electrotransferred onto polyvinylidene difluoride membranes (Millipore, Billerica, MA, USA), and analyzed using specified antibodies with an ECL detection system (GE Healthcare, Chicago, IL, USA).

### Real-time quantitative reverse transcriptase PCR

Total RNA was extracted from the indicated cell lines using an RNeasy mini kit (Qiagen, Hilden, Germany) according to the manufacturer’s instructions. RNA was reverse-transcribed to cDNA with PrimeScript™ RT Master Mix (Takara, Shiga, Japan). Quantitative real-time PCR was performed using the TB Green™ Premix Ex Taq™ II (Takara). Primer sequences are shown in Table 3. Relative amounts of mRNA were calculated from the threshold cycle number using glyceraldehyde 3-phosphate dehydrogenase (GAPDH) as an endogenous control. All experiments were performed in triplicate and the values were averaged.

**Table 3:** Primer sequences used for qPCR experiments.

Gene	Sequence	
E-cadherin	Forward	AAG AAG CTG GCT GAC ATG TAC GGA
(*CDH1*)	Reverse	CCA CCA GCA ACT GAT TTC TGC AT
Vimentin	Forward	AGA ACC TGC AGG AGG CAG AAG AAT
(*VIM*)	Reverse	TTC CAT TTC ACG CAT CTG GCG TT
PD-L1	Forward	AGC CCT CAG CCT GAC ATG TC
(*CD274*)	Reverse	GGT GCC GAC TAC AAG CGA AT
GAPDH	Forward	TGC ACC ACC AAC TGC TTA GC
	Reverse	GGC ATG GAC TGT GGT CAT GAG

### Statistical analysis

To test the prognostic significance, only gene expression data with available survival data were used. Prognostic significance was estimated by the Kaplan-Meier method and log-rank test between two predicted subgroups. Univariate and multivariate Cox proportional hazards regression analysis was performed to evaluate independent prognostic factors associated with survival. Pearson’s correlation was used for correlation analysis and Fisher’s exact test was used to assess the frequency difference of somatic mutation. All statistical analyses were performed using the R language program (http://www.r-project.org).

## Supplementary information

Supplementary information.
