# Supplementary material for: Epithelial-mesenchymal transition gene signature is associated with prognosis and tumor microenvironment in head and neck squamous cell carcinoma
Source: Sci Rep. 2020 Feb 27;10:3652. doi: 10.1038/s41598-020-60707-x (PMC7046610; doi:10.1038/s41598-020-60707-x)

Supplementary Information

Epithelial-mesenchymal transition gene signature is associated with prognosis and tumor microenvironment in head and neck squamous cell carcinoma

Ah Ra Jung[1+], Chan-Hun Jung[1+], Joo Kyung Noh[2], Young Chan Lee[1], Young-Gyu Eun[1*]

[1]Department of Otolaryngology - Head & Neck Surgery, School of Medicine, Kyung Hee University, Seoul, Korea

[2]Department of Biomedical Science and Technology, Graduate School, Kyung Hee University, Seoul, Korea

[+]These authors contributed equally to this study.

***Corresponding author:** Young-Gyu Eun, E-mail: ygeun@khu.ac.kr

Supplementary Table S1. The EMT signature genes

**Gene symbol**	**correlation coefficient with gene expression of *CDH1***	**correlation coefficient with gene expression of *VIM***	**correlation coefficient with gene expression of *CDH2***	**correlation coefficient with gene expression of *FN1***	***p*-value**
CDH1	1				NA
DSP	0.715				1.04959E-81
PKP1	0.714				1.68453E-81
MAP7	0.704				4.01876E-78
ESRP1	0.774				1.1383E-103
KRT6A	0.640				1.00919E-60
C1orf116	0.621				2.69365E-56
SERPINB5	0.608				2.27987E-53
LASS3	0.604				1.79357E-52
LAD1	0.685				1.74058E-72
PERP	0.612				2.81884E-54
JUP	0.646				4.05629E-62
DSG3	0.654				4.00587E-64
DSC3	0.605				9.63006E-53
OVOL1	0.651				3.09526E-63
GRHL2	0.644				1.44519E-61

ERBB3	0.605	1.06497E-52
MARVELD3	0.616	5.51337E-55
EVPL	0.643	1.95081E-61
TRIM29	0.680	4.86046E-71
FAM83B	0.618	1.6913E-55
ESRP2	0.661	5.47739E-66
CDS1	0.666	3.79693E-67
EPN3	0.606	5.70893E-53
EPHA1	0.601	7.00609E-52
KLF5	0.606	5.73733E-53
ABLIM1	0.618	1.8118E-55
PPL	0.646	3.72067E-62
GRHL3	0.691	2.25004E-74
HAS3	0.623	1.40427E-56
ZNF750	0.664	7.93184E-67
A2ML1	0.626	3.0313E-57
PRRG4	0.627	1.74849E-57
MAL2	0.651	2.39857E-63
RAB25	0.650	3.76869E-63
TTC22	0.615	7.8741E-55

IRF6	0.624				8.22508E-57
GRHL1	0.699				9.53222E-77
VIM		1			NA
ANXA6		0.763			2.7572E-99
LIX1L		0.724			1.3177E-84
HLX		0.710			4.06598E-80
SYT11		0.706			5.93141E-79
GNAI2		0.706			8.54983E-79
FYN		0.731			2.79586E-87
ATP10A		0.717			2.19778E-82
CNRIP1		0.725			5.77421E-85
EMILIN1		0.730			6.08807E-87
DAB2		0.735			1.51914E-88
OLFML2B		0.778			1.7081E-105
COL6A1		0.721			7.96813E-84
ZEB2		0.742			4.06176E-91
COL6A2		0.750			5.94027E-94
ST3GAL2		0.758			4.20545E-97
OLFML3		0.756			2.43112E-96
ACVRL1		0.702			1.34816E-77

CMTM3	0.725			6.65313E-85
PMP22	0.746			1.14563E-92
PCOLCE	0.751			2.50175E-94
GPR124	0.707			3.63533E-79
TIMP2	0.709			8.15841E-80
LAMA4	0.714			2.50937E-81
CDH2		1		NA
DACT1		0.708		2.42451E-79
FN1			1	NA
FSTL1			0.815	1.7796E-123
FAP			0.809	1.3555E-120
CALD1			0.808	4.766E-120
COL5A1			0.843	5.4019E-140
VCAN			0.823	3.635E-128
COL5A2			0.829	1.3833E-131
GLT8D2			0.807	2.9154E-119
POSTN			0.853	9.5235E-147
COL1A1			0.850	7.4847E-145
ADAM12			0.830	3.0152E-132
COL6A3			0.830	5.032E-132

SPARC	0.819	1.1234E-125
ZNF469	0.811	2.2673E-121
COL1A2	0.840	2.9374E-138
NID2	0.847	9.8379E-143
COL3A1	0.838	3.2086E-137
PDGFRB	0.802	5.2039E-117

Abbreviations: NA, not available.

Supplementary Table S2. Association of the EMT signature with other subtypes of HNSCC.

	**Mes**	**Epi**	***p*-value**
Gender			0.702
Male	267	247	
Female	86	73	
Age			0.305
<60 years	167	165	
>60 years	186	155	
Tumor site			0.074
Oral cavity	189	156	
Oropharynx	69	79	
Larynx	66	71	
Hypopharynx	26	13	
Primary tumor			0.851
T1	42	33	
T2	100	98	
T3	75	70	
T4	136	119	
Regional lymph node			0.138
N (-)	152	157	
N (+)	201	163	
Stage			0.383
I	17	18	
II	65	57	
III	56	66	
IV	215	179	
HPV status			0.187
Positive	46	51	
Negative	228	183	
Tobacco use			0.995
Never	74	67	

Yes	272	251	
Alcohol use			0.171
Never	79	87	
Yes	270	229	

Supplementary Table S3. Comparison of somatic mutation frequencies between the Mesenchymal and Epithelial groups.

		Mes	Epi	Fisher's *p*-value
EGFR	MUT	15	4	1.89E-03*
	WT	242	232	
HRAS	MUT	11	16	0.240
	WT	246	220	
KRAS	MUT	1	0	1
	WT	256	236	
PIK3CA	MUT	45	45	0.726
	WT	212	191	
PTEN	MUT	6	8	0.591
	WT	251	228	
TP53	MUT	200	151	0.001*
	WT	57	85	

Supplementary Table S4. Top significant KEGG pathways associated with EMT signature genes.

KEGG pathway	Count	Molecules	*p*-value
ECM-receptor interaction	10	COL1A1, COL1A2, COL3A1, COL5A1, COL5A2, COL6A1, COL6A2, COL6A3, FN1, LAMA4	4.3e-11*
Focal adhesion	12	FYN, COL1A1, COL1A2, COL3A1, COL5A1, COL5A2, COL6A1, COL6A2, COL6A3, FN1, LAMA4, PDGFRB	2.7e-10*
Protein digestion and absorption	8	COL3A1, COL5A1, COL5A2, COL6A1, COL6A2, COL6A3, COL1A1, COL1A2	5.3e-8*
PI3K-Akt signaling pathway	11	COL1A1, COL1A2, COL3A1, COL5A1, COL5A2, COL6A1, COL6A2, COL6A3, FN1, LAMA4, PDGFRB	7.2e-7*
Amoebiasis	5	COL3A1, FN1, LAMA4, COL1A1, COL1A2	4.0e-6*
Platelet activation	5	COLA31, GNAI2, FYN, COL1A1, COL1A2	1.3e-5*
Axon guidance	4	EPHA1, FYN, ABLIM1, GNAI2	0.016*
Pathways in cancer	6	GNAI2, CDH1, FN1, JUP, LAMA4, PDGFRB	0.022*
MicroRNAs in cancer	5	ERBB3, PDGFRB, SERPINB5, VIM, ZEB2	0.03*
Arrhythmogenic right ventricular cardiomyopathy (ARVC)	3	CDH2, DSP, JUP	0.035*

Supplementary Figure S1. Full length uncropped western blotting images used to generate Figure 5.

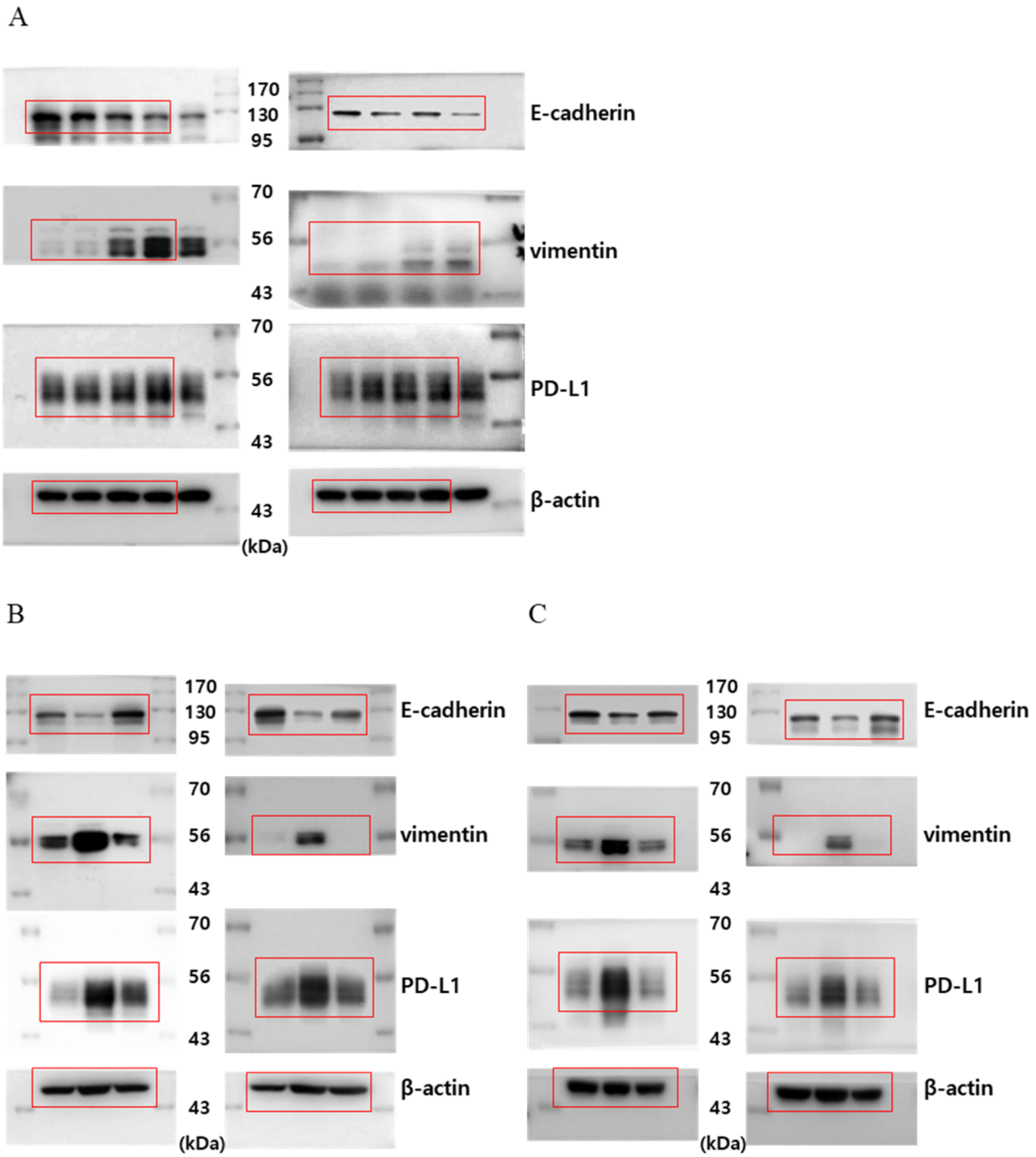

Supplement: Supplementary file 1 — Supplementary information. [file 41598_2020_60707_MOESM1_ESM.pdf]
